# Temperature Sensitivity as a Microbial Trait Using Parameters from Macromolecular Rate Theory

**DOI:** 10.3389/fmicb.2016.01821

**Published:** 2016-11-17

**Authors:** Charlotte J. Alster, Peter Baas, Matthew D. Wallenstein, Nels G. Johnson, Joseph C. von Fischer

**Affiliations:** ^1^Department of Biology, Colorado State University, Fort CollinsCO, USA; ^2^Graduate Degree Program in Ecology, Colorado State University, Fort CollinsCO, USA; ^3^Natural Resource Ecology Laboratory, Colorado State University, Fort CollinsCO, USA; ^4^Department of Ecosystem Science and Sustainability, Colorado State University, Fort CollinsCO, USA; ^5^National Institute for Mathematical and Biological Synthesis, University of Tennessee, KnoxvilleTN, USA

**Keywords:** macromolecular rate theory, MMRT, activation energy, Q10, temperature optimum, extracellular enzymes, microbial isolates, trait-based ecology

## Abstract

The activity of soil microbial extracellular enzymes is strongly controlled by temperature, yet the degree to which temperature sensitivity varies by microbe and enzyme type is unclear. Such information would allow soil microbial enzymes to be incorporated in a traits-based framework to improve prediction of ecosystem response to global change. If temperature sensitivity varies for specific soil enzymes, then determining the underlying causes of variation in temperature sensitivity of these enzymes will provide fundamental insights for predicting nutrient dynamics belowground. In this study, we characterized how both microbial taxonomic variation as well as substrate type affects temperature sensitivity. We measured β-glucosidase, leucine aminopeptidase, and phosphatase activities at six temperatures: 4, 11, 25, 35, 45, and 60°C, for seven different soil microbial isolates. To calculate temperature sensitivity, we employed two models, Arrhenius, which predicts an exponential increase in reaction rate with temperature, and Macromolecular Rate Theory (MMRT), which predicts rate to peak and then decline as temperature increases. We found MMRT provided a more accurate fit and allowed for more nuanced interpretation of temperature sensitivity in all of the enzyme × isolate combinations tested. Our results revealed that both the enzyme type and soil isolate type explain variation in parameters associated with temperature sensitivity. Because we found temperature sensitivity to be an inherent and variable property of an enzyme, we argue that it can be incorporated as a microbial functional trait, but only when using the MMRT definition of temperature sensitivity. We show that the Arrhenius metrics of temperature sensitivity are overly sensitive to test conditions, with activation energy changing depending on the temperature range it was calculated within. Thus, we propose the use of the MMRT definition of temperature sensitivity for accurate interpretation of temperature sensitivity of soil microbial enzymes.

## Introduction

The activities of extracellular enzymes, a rate-limiting step in decomposition and important component in biogeochemical cycles (Burns and Dick, 2002), are strongly controlled by *in situ* temperatures (Davidson and Janssens, 2006; Wallenstein et al., 2011). Although the importance of enzyme temperature sensitivity is widely recognized, the degree to which temperature sensitivity is an inherent property of the enzymes vs. a response to environmental conditions (Davidson and Janssens, 2006) is largely unknown. It has been difficult to parse the mechanisms underlying observations of enzyme temperature responses in part because assays are typically conducted at the community level where the contribution of isoenzymes produced by individual taxa cannot be isolated (Bradford, 2013; Karhu et al., 2014). In addition, relative and absolute measures of temperature sensitivity using the same, simple models often produce contradictory results (Sierra, 2012). Understanding if these soil extracellular enzymes have inherent temperature sensitivity is critical for accurate predictions of soil carbon (C) and other nutrient dynamics in changing environments. In this study we attempt to determine the degree to which soil enzymes are responsive to temperature. In addition, we focus on clearing up some of the definitional confusions regarding temperature sensitivity in soils.

Over most of the last decade, the debate on if and how temperature sensitivity differs among enzymes has used parameters from two models: the *Q*_10_ temperature coefficient and activation energy, *E*_A_, derived from the Arrhenius equation. *Q*_10_ is a unitless measure of the change in rate with a 10-degree increase in temperature,

(1) $$Q_{10}={\left(\frac{R_{2}}{R_{1}}\right)}^{10/(T_{2}-T_{1})},$$

where *R* is reaction rate and *T* is temperature. The *Q*_10_ of biological systems is generally thought to be ∼2 or 3, although it has been found to be substantially higher in some soils and enzymes (Lloyd and Taylor, 1994; Chapin and Matson, 2011; Elias et al., 2014). The Arrhenius equation describes temperature response as

(2) $$\text{In}(k)=\text{In}(A)-\frac{E_{A}}{RT},$$

where *k* is the reaction rate constant, *A* is a pre-exponential factor, *E*_A_ is the activation energy, *R* is the universal gas constant, and *T* is temperature. According to the Arrhenius model, temperature sensitivity is compared using *E*_A_ as the parameter of interest instead of or in addition to *Q*_10_. Reactions with higher *Q*_10_ values require a larger “push” or activation energy (*E*_A_) to initiate the reaction (Davidson and Janssens, 2006).

Previously, studies have drawn varying conclusions about how *Q*_10_ and *E*_A_ vary with enzyme structure and function. From an evolutionary perspective, selection should generate an adaptive fit of enzyme kinetics to their thermal environment (Allison et al., 2011; Bradford, 2013). For example, thermophilic enzymes tend to have increased conformational rigidity (Zavodszky et al., 1998), while psychrophilic enzymes have improved flexibility, particularly at the active site (Feller, 2003; Struvay and Feller, 2012), impacting temperature sensitivity. Many soil studies have observed significant differences among *Q*_10_ and *E*_A_ values at a range of spatial and temporal scales (Koch et al., 2007; Trasar-Cepeda et al., 2007; Wallenstein et al., 2009; Brzostek and Finzi, 2012; Steinweg et al., 2013). Despite the predicted and observed differences between these different types of enzymes and isoenzymes, an analysis conducted by (Elias et al., 2014) found no statistical difference across enzyme classes in *Q*_10_ values from 150 enzymatic reactions.

One reason why patterns in *Q*_10_ and *E*_A_ are not easily explained is that they may not be the most appropriate parameters to evaluate temperature sensitivity from soil microbial enzymes (Schipper et al., 2014). It has long been recognized that Arrhenius and *Q*_10_ do not always accurately describe the relationship between temperature and reaction rates in soil systems (Lloyd and Taylor, 1994), yet they have continued to be used out of convenience, convention, or perhaps due to lack of a better alternative model. The most conspicuous disparity between these models is the empirical data commonly observed showing negative curvature (i.e., a concave-down parabolic response) in rate vs. temperature, which is not explained by either *Q*_10_ or *E*_A_. This negative curvature is typically ascribed to enzyme denaturation even though the pattern is sometimes observed at relatively low temperatures. We hypothesize that this negative curvature causes estimated *Q*_10_ and *E*_A_ values to vary with the temperature range where they are measured, thus making them more phenomenological parameters than fundamental system properties (Pawar et al., 2016). A second issue is that thermodynamic principles indicate that the Arrhenius models are missing a key term when applied in biological systems: for large macromolecules like enzymes, it is not appropriate to assume that the transition state of Gibbs Free Energy (Δ*G*^‡^) is constant across temperatures (Hobbs et al., 2013).

A relatively new model, Macromolecular Rate Theory (MMRT), accounts for both the physical and biological components of reaction rate with temperature (Hobbs et al., 2013). MMRT is defined as,

(3) $$\text{In}(k)=\text{In}(\frac{k_{\text{B}}T}{h})-\frac{\Delta H_{\text{T0}}^{‡}+\Delta C_{\text{p}}^{‡}(T-T_{0})}{RT}+\frac{\Delta S_{{\text{T}}_{\text{0}}}^{‡}+\Delta C_{\text{p}}^{‡}(InT-InT_{0})}{R}$$

where *k* is the rate constant, *k*_B_ is Boltzmann’s constant, *h* is Planck’s constant, *R* is the universal gas constant, *T* is temperature, *H* is enthalpy, *S* is entropy, *C*_P_ is heat capacity, and ‡ indicates that it is the transition state. Thus, we propose that the idea of “temperature sensitivity” when described by MMRT emerges as three fundamental components: the heat capacity of the enzyme ($C_{p}^{‡}$), the temperature optimum (*T*_opt_), and the point of maximum temperature sensitivity (TS_max_). The heat capacity of the enzyme describes the degree of curvature in the parabolic response of reaction rate with temperature; more parabolic curves have larger, negative values of Δ$C_{p}^{‡}$ that arise when enzymes are more rigid at the transition state. The temperature optima denote the point at which the reaction rate is greatest, with lower reaction rates at higher temperatures not necessarily indicating enzyme denaturation. The point of maximum temperature sensitivity is calculated from the first derivative of *k* with temperature (*dk/dT*), and indicates the temperature where the rate of change is greatest.

Our proposed concept of temperature sensitivity could enable use of traits-based approaches for understanding how community-level patterns in temperature sensitivity are related to thermal responses of the many isoenzymes produced by diverse microbes. Under this definition of temperature sensitivity, Δ$C_{p}^{‡}$, *T*_opt_, and TS_max_ are measurable properties of individual organisms, or enzymes from organisms, and one that clearly would influence organismal performance, falling under the ecological definition of a functional trait (McGill et al., 2006). Including temperature sensitivity in a traits-based framework could enable the linking of the microbial community with soil ecosystem functioning (Green et al., 2008), as well as allow for a stronger quantitative approach to integrate temperature sensitivity into models for improved predictive power (Webb et al., 2010).

If temperature sensitivities of enzymes are in fact traits and exhibit variation based on genetic and environmental variation, we hypothesize that different enzymes will demonstrate distinct temperature sensitivities, as defined by the terms Δ$C_{p}^{‡}$, *T*_opt_, and TS_max_. Because the difference in Δ$C_{p}^{‡}$ is impacted by the physical flexibility of the enzyme (Hobbs et al., 2013), which we hypothesize is a result of genetic variation among communities and/or from interaction with substrate type, we predict that temperature sensitivity of soil extracellular enzymes will vary by the microbe from which the enzyme was derived or by the enzyme type. Because most enzymes are substrate specific, different enzyme-substrate complexes can have a wide range of Δ$C_{p}^{‡}$ values. Moreover, different microbes produce different isoenzymes, so temperature sensitivity may also vary among microbes.

We measured extracellular enzyme activity from seven soil isolates and three different enzymes at six temperatures, in order to advance the study of temperature sensitivity as an intrinsic microbial trait. In a previous study (Alster et al., 2016), we found that temperature sensitivity varied among soil microbial communities. We applied both Arrhenius and MMRT to our data to compare the effectiveness of each of these models and demonstrate how Arrhenius estimates of temperature sensitivity may not be sufficient, even within *in situ* representative temperature ranges.

## Materials and Methods

### Experimental Design

Extracellular enzymatic assays were performed for three enzymes, β-glucosidase (BG), leucine aminopeptidase (LAP), and phosphatase (PHOS) on seven soil isolates, each from a different genera—*Acinetobacter, Bacillus, Citrobacter, Comamonas, Enterobacter, Flaviobacterium*, and *Pseudomonas*. The isolates were derived either from soil or worm castings and kept at -80°C with 20% glycerol until use. The isolates were revived from storage and grown in nutrient broth over a 2–3 days period at 25°C. We added 3-(*N*-morpholino)propanesulfonic acid buffer to maintain a pH of 7.2, which is the same pH as the microbes were originally isolated at. Before we began the enzyme assays, the isolate solution was plated on nutrient broth agar and total incubation time was determined by when cultures reached between 10^5^–10^7^ colony-forming units per mL.

The isolates were incubated in 96-well microplates with substrates at six temperatures: 4, 11, 25, 35, 45, and 60°C. We chose a large initial temperature range in order to capture the most accurate temperature response curve. The enzyme assay was modified from Bell et al. (2013). Forty microliters of 200 mM fluorometric substrate—4-MUB-β-D-glucopyranoside for BG, L-leucine-7-amido-4-methylcoumarin hydrochloride for LAP, and 4-MUB phosphate for PHOS—was added to 160 μL of a 1 isolate mixture: 15 acetate buffer solution. For each isolate × substrate combination, there were eight replicates for each temperature (7 isolates × 3 enzymes × 6 temperatures × 8 replicates). Standards ranging from 2.5 to 100 μM were used to calibrate the enzyme activity from each enzyme. 4-methylumbelliferone (MUB) was used to calibrate BG and PHOS and 7-amino-4-methylcoumarin (MUC) was used to calibrate LAP. The plates were incubated between 1 and 23 h depending on the temperature and scanned on a Tecan Infinite M200 plate reader at optimal florescence as determined by the MUB and MUC standards. Reaction rates were linear regardless of incubation time, as determined by preliminary experiments. The MUB and MUC standard curves were used to calculate the raw florescence of the samples using the slope and y-intercept, as described in Bell et al. (2013) and converted into units of nmol activity L culture^-1^ hour^-1^, so that samples were comparable across temperatures with varying incubation times.

### Calculating Temperature Sensitivity

In order to quantitatively characterize temperature responses of each of the 21 isolate × enzyme combinations, we plotted the natural log of the reaction rate against temperature and fitted both the Arrhenius and MMRT equations using an analytic Gauss-Newton for Arrhenius and a numerical Gauss-Newton for MMRT in JMP Pro 11 (Schipper et al., 2014; Alster et al., 2016). Parameters *E*_A_ and Δ$C_{p}^{‡}$, along with their uncertainty, were reported by the software. The optimum temperature (*T*_opt_) and point of maximum temperature sensitivity (TS_max_) from the MMRT curve fits were calculated by taking the derivative of the MMRT equation with respect to temperature (Alster et al., 2016). We used a Monte Carlo Simulation to estimate the standard error for *T*_opt_ and TS_max_.

Analysis of variances (ANOVA) were performed using the software R version 3.2.1 (R Core Team, 2015) to determine the relative importance of substrate type and species type in explaining variation in the parameters from each of the models (Δ$C_{p}^{‡}$, Δ$S_{\mathrm{T0}}^{‡}$, Δ$H_{\mathrm{T0}}^{‡}$, *E*_A_, and *A*) as well as for *T*_opt_ and TS_max_. We also used R to run pairwise comparisons with a Holm multiple testing adjustment to examine differences between each of the model parameters and *T*_opt_ and TS_max_ from each of the isolate × enzyme combinations. Differences in Δ$C_{p}^{‡}$ were calculated using a two-sampled approximate *Z*-test.

### Comparison of MMRT and Arrhenius Equations

We used adjusted *R*^2^ and Akaike information criterion corrected for a finite sample size (AICc) to determine the most parsimonious model between the Arrhenius and MMRT model fits for the full temperature range (4–60°C). Additionally, we re-ran the Arrhenius model fit for each isolate × enzyme combination, but only using temperatures 4–25 and 4–35°C to evaluate if, under more biologically relevant temperatures, the Arrhenius model fits were accurate predictors of reaction rate. To assess this, we calculated the percentage that each of the three models (MMRT from 4 to 60°C, Arrhenius from 4 to 35°C, and Arrhenius from 4 to 25°C) over or underestimated the reaction rate as compared to the actual experimental values and conducted corresponding lack-of-fit (LOF) tests. The importance of each of these models for predicting the percent error was examined with a linear model and tested with an ANOVA using the lmerTest package in R (Kuznetsova et al., 2014).

## Results

### Temperature Sensitivity Differs for Isolate × Enzyme Combinations

**FIGURE 1:**
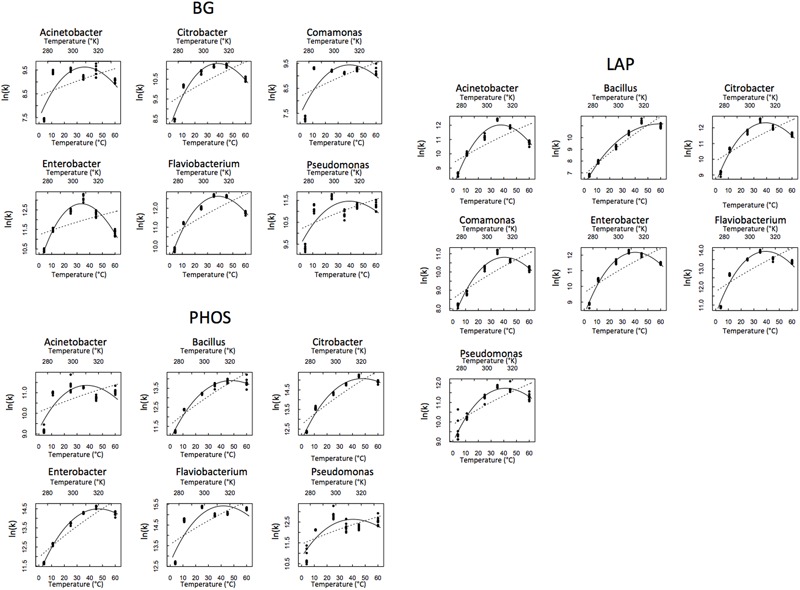
**Temperature response of each isolate and enzyme combination.** Macromolecular Rate Theory (MMRT) is represented by the solid line and Arrhenius is represented by the dashed line.

Out of the 21 isolate × enzyme combinations we tested, we present here the results from the 19 that worked. BG activity in *Bacillus* and PHOS activity in *Comamonas* were below detection limits. Thus, these two combinations were eliminated from the analysis. We plotted the reactions rates of the remaining 19 isolate × enzyme combinations vs. temperature and fit both the MMRT and Arrhenius equations to the data (**Figure 1**). These model fits for MMRT give temperature sensitivity parameters Δ$C_{p}^{‡}$, *T*_opt_ and TS_max_, while Arrhenius gives *E*_A_ as a parameter.

**FIGURE 2:**
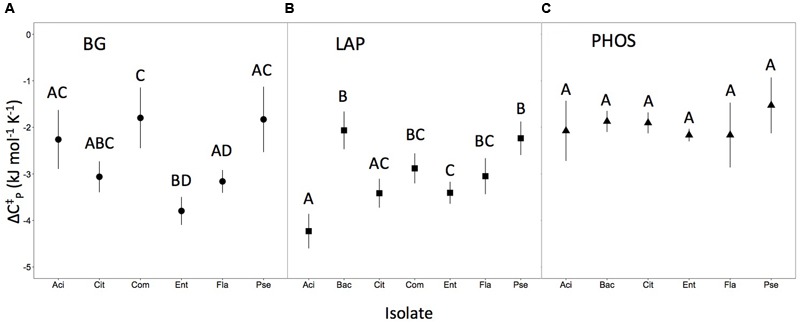
**Heat capacity for each isolate and enzyme combination.** Error bars (±2SE) represent uncertainty in the model fit. Letters represent significant differences (*P* < 0.05) between isolates of the same enzyme (i.e., within panels), but not across the different enzyme types (i.e., not between panels). Panels correspond to heat capacity responses for **(A)** isolates for the BG enzyme, **(B)** isolates for the LAP enzyme, and **(C)** isolates for the PHOS enzymes.

We found that Δ$C_{p}^{‡}$ differed for some, but not all of the isolate × enzyme combinations. The Δ$C_{p}^{‡}$ differed among microbial isolates in BG and LAP enzymes (*P* < 0.05; **Figures 2A,B**). However, in PHOS, the isolates did not differ in Δ$C_{p}^{‡}$ (*P* > 0.05; **Figure 2C**). When comparing if Δ$C_{p}^{‡}$ differed between the same isolate for different enzymes we found significant differences in Δ$C_{p}^{‡}$ for *Acinetobacter, Citrobacter*, and *Enterobacter* (*P* < 0.05), but not for *Bacillus, Comamonas, Flaviobacterium*, or *Pseudomonas*. Patterns in statistical differences were identical for Δ$C_{p}^{‡}$, Δ*S*^‡^, and Δ*H*^‡^. Overall 70.9% of variation in Δ$C_{p}^{‡}$ was explained by the microbial isolate type, compared with 29.1% of the variation explained by the enzyme type.

The temperature optima (*T*_opt_) and point of maximum temperature sensitivity (TS_max_) also varied with the isolate × enzyme combinations (**Table 1**). Despite the 25°C conditions used during initial culturing of the inoculum isolates, *T*_opt_ ranged from 33.5°C in BG for the *Enterobacter* isolate to 60.7°C in LAP for the *Bacillus* isolate and TS_max_ ranged from 18.2°C in BG for the *Acinetobacter* isolate to 40.3°C in LAP for the *Bacillus* isolate. Pooling the *T*_opt_ and TS_max_ values across the same microbial isolate and enzyme type, we found differences for some of the values across both microbial isolate and enzyme type (*P* < 0.05). While differences between these pooled values for *T*_opt_ and TS_max_ were similar, they were not identical. Furthermore, similar to Δ$C_{p}^{‡}$, variation in *T*_opt_ and TS_max_ are best explained by the microbial isolate type, with SS values of 86.7 and 80.0%, respectively.

**Table 1:** Mean temperature optima (*T*_opt_) and point of maximum temperature sensitivity (TS_max_) for each isolate and enzyme combination ± SEM.

	BG		LAP		PHOS		Pooled estimate	
	*T*_opt_	TS_max_	*T*_opt_	TS_max_	*T*_opt_	TS_max_	*T*_opt_	TS_max_
*Acinetobacter*	36.0 ± 1.9	18.2 ± 2.4	37.7 ± 0.6	24.45 ± 0.6	37.55 ± 2.1	18.85 ± 2.6	37.1 ± 2.9	20.5 ± 3.6
*Bacillus*	NA	NA	60.7 ± 2.5	40.25 ± 1.5	48.05 ± 1	27.95 ± 0.7	54.2 ± 3.3	34.1 ± 3.0
*Citrobacter*	38.1 ± 0.7	22.6 ± 0.8	39.7 ± 0.6	24.55 ± 0.6	47.95 ± 0.9	27.95 ± 0.7	41.7 ± 1.3	25.0 ± 1.2
*Comamonas*	39.1 ± 2.5	19.0 ± 3.1	40.6 ± 0.7	24.55 ± 0.8	NA	NA	39.8 ± 2.6	21.8 ± 3.2
*Enterobacter*	33.5 ± 0.6	19.8 ± 0.7	40.0 ± 0.4	25.25 ± 0.5	46.85 ± 0.5	28.25 ± 0.4	40.1 ± 0.9	24.4 ± 0.9
*Flaviobacter*	38.4 ± 0.5	23.2 ± 0.6	39.4 ± 0.8	23.85 ± 0.9	41.85 ± 2.2	23.25 ± 2.3	39.9 ± 2.4	23.4 ± 2.5
*Pseudomonas*	39.0 ± 2.6	19.1 ± 3.2	41.4 ± 1.1	23.25 ± 1.1	40.45 ± 2.7	18.55 ± 3.1	40.3 ± 3.9	20.3 ± 4.6
**Pooled estimate**	37.3 ± 4.2	20.3 ± 5.2	42.6 ± 3.1	26.6 ± 2.4	43.8 ± 4.3	24.1 ± 4.8	–	–

*Pooled estimates are averages either across enzymes for a given isolate or across isolates for a given enzyme.*

The three metrics of temperature sensitivity, Δ$C_{p}^{‡}$, *T*_opt_ and TS_max_, each have unique statistical patterns of similarity across inocula × enzyme combinations. In this paper, we do not deeply examine the basis for groupings but focus instead on identifying if patterns of similarity and difference exist or if all enzymes behave similarly. For conciseness, we illustrate only patterns of differences for Δ$C_{p}^{‡}$ in **Figure 2**, and provide *T*_opt_ and TS_max_ findings in **Table 1**. For PHOS we found no differences in Δ$C_{p}^{‡}$ among the different microbial isolates (**Figure 2**). For TS_max_ of PHOS there were no differences, but for *T*_opt_ we found several significant differences among isolates (**Table 1**). Likewise for BG, there were quite a few differences among isolates in Δ$C_{p}^{‡}$ (**Figure 2**). However, there were no significant differences between different microbial isolates in TS_max_ for BG, and while there are differences in *T*_opt_, they are not the same as the differences identified for Δ$C_{p}^{‡}$. Interestingly, patterns in significant differences among isolates for LAP are the same for *T*_opt_ and TS_max_, but show a different pattern for Δ$C_{p}^{‡}$. Some of these patterns likely emerge because *T*_opt_ and TS_max_ are positively correlated (*R*^2^ = 0.84), while Δ$C_{p}^{‡}$ does not correlated with *T*_opt_ or TS_max_ (*R*^2^ = 0.23 and *R*^2^ = 0.01, respectively).

### MMRT Provides Better Statistical Fit than Arrhenius

**FIGURE 3:**
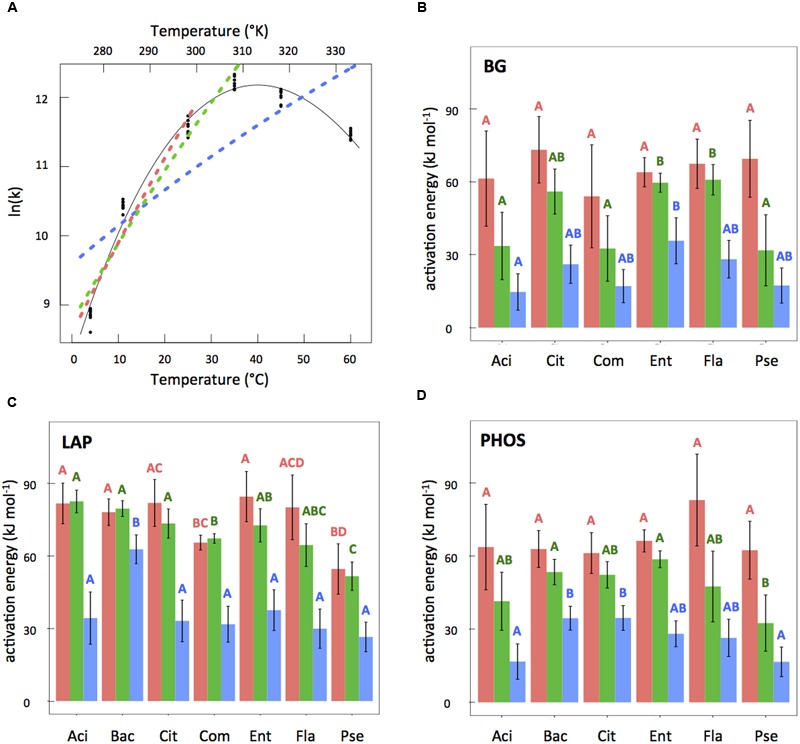
**(A)** Example temperature response plot (LAP for *Enterobacter*) showing fits for MMRT, Arrhenius with temperature range 4–60°C (blue, dashed line), Arrhenius with temperature range 4–35°C (green, dashed line), and Arrhenius with temperature range 4–25°C (pink, dashed line). **(B–D)** Activation energy estimates from the three Arrhenius fits with bars corresponding to Arrhenius 4–25°C (pink), Arrhenius 4–35°C (green), and Arrhenius with temperature range 4–60°C (blue). Error bars (±2SE) represent uncertainty in the model fit. Letters represent significant differences (*P* < 0.05) between different isolates for the same Arrhenius fit (i.e., 4–25, 4–35, or 4–60°C, not between different fits for the same isolate) and within the same enzyme types (i.e., not between panels). Only compare same colors within the same panels.

For the temperature range of 4–60°C, we found that MMRT gave vastly superior fits to the data as compared to Arrhenius according to both AICc and R^2^ criteria (Supplementary Table S1). MMRT was also superior when the Arrhenius model was fit to the more linear part of the temperature range (4–35 and 4–25°C) for 13 of the 19 isolate × enzyme combinations (see example, **Figure 3A**; Supplementary Table S1). For the six combinations where MMRT was not superior, AICc analysis found MMRT and Arrhenius to have equivalent explanatory power; in no case was Arrhenius the superior model. Phosphatase was the only enzyme where MMRT was significantly better in all isolates tested.

Despite the statistically improved fit of MMRT as compared to Arrhenius, when comparing the overall error produced from each of the model predictions, the results were less striking. We found 3.8% of the total variation was explained by LOF from the model in MMRT compared with 6.2% for Arrhenius from 4 to 35°C and 10.3% for Arrhenius from 4 to 25°C. This means that by using Arrhenius instead of MMRT we are introducing 1.6 and 2.7 times more error into our predictions for the Arrhenius 4–35 and 4–25°C models, respectively. ANOVA results for the percent error of the models compared to the experimentally observed results at 4, 11, 25, and 35°C, revealed a significant difference between MMRT and the Arrhenius 4–25°C model (*P* ≤ 0.05) and between the two Arrhenius models (*P* = 0.02). However, there was no significant difference in percent error between MMRT and the Arrhenius 4–35°C model fits when compared to original activity values at 4, 11, 25, and 35°C.

### Comparison of Activation Energy Values

To examine the value of using activation energy as a trait, we compared activation energies derived from different temperature ranges to see if they vary. In a 3-way ANOVA examining the *E*_A_ values from all three of the Arrhenius model temperature ranges tested, we found that the temperature range explained 68.5% of variation in the data, compared to 12.7% explained by enzyme type and 11.0% explained by isolate type. Overall, we found that as the temperature range increased *E*_A_ values decreased (**Figures 3B–D**). Not only did the absolute values of *E*_A_ vary based on temperature range, but the relative *E*_A_ values also differ (**Figures 3B–D**) leading to different groupings of similarity among assays. In comparisons of *E*_A_ values for the different inocula × enzyme combinations that shared either the same enzyme or same isolate, 25% of the relationships changed between the Arrhenius 4–25 and 4–35°C estimates, and 36.8% of the relationships changed between the Arrhenius 4–35 and 4–60°C estimates. A similar analysis capturing different temperature ranges was not needed for MMRT since MMRT captures the peak.

## Discussion

### Heat Capacity Differs Significantly among Enzymes

This study advances efforts to understand how temperature sensitivity of extracellular enzymes varies by substrate and isolate type. Such an effect has long been speculated, based on assays conducted at the community level with whole soils (e.g., Trasar-Cepeda et al., 2007; Steinweg et al., 2013). We found that Δ$C_{p}^{‡}$ differed significantly among isolates for the BG and LAP enzymes, across the different isolates measured (**Figure 2**). Furthermore, heat capacity of different enzymes varied within the same isolate for three out of the seven isolates measured. While this study was not designed to elucidate the mechanisms behind why heat capacity varied between some enzymes and isolates but not others, here we provide a few possible explanations. One broad explanation for why we see these differences is that microbes adapt to their environment and more efficient enzymes are selected for in accordance to the thermodynamic conditions in that environment (Bradford, 2013); thus, microbes will adapt to produce isoenzymes with varying degrees of flexibility and consequently different heat capacities values depending on what is most advantageous for the microbial cell’s survival. The idea that isoenzymes have distinct temperature sensitivities is not particularly groundbreaking if comparing enzymes derived from thermophilic, psychrophilic, and mesophilic conditions (Zavodszky et al., 1998; Lonhienne et al., 2000; Feller and Gerday, 2003) or even across the same soil microbial community throughout seasons (Koch et al., 2007; Trasar-Cepeda et al., 2007; Wallenstein et al., 2009). However, in this study all of the isolates measured were derived from a mesophilic environment and raised in culture at the same temperature (25°C). Thus, we found that temperature sensitivity varies even among organisms raised under the same temperature conditions.

In contrast to BG and LAP, the heat capacity of PHOS was invariant with isolate type, suggesting that perhaps this enzyme did not undergo a similar type of adaptation over evolutionary history or that there are simply fewer isoenzymes. Although the PHOS enzyme is ubiquitous across different types of organisms, the genomic region encoding for the active site is highly conserved and fairly homologous across plants, animals, and bacteria, at least for the acidic version of the enzyme (Anand and Srivastava, 2012). As opposed to aminopeptidases in which relatively few homologies have been observed despite their high abundance (Taylor, 1993), gene conservation of PHOS might explain the lack of variation in heat capacity. Thermodynamic constraints of the enzyme or active site may also limit adaptation if there is a fundamental evolutionary tradeoff between the structure and function of the enzyme that is specific to the catalytic properties of PHOS (Bradford, 2013).

It is also worth noting that because these estimates of heat capacity were not necessarily of individual enzymes, but of the all of the isoenzymes produced by the isolates under the incubation conditions of this experiment. While it is unclear if multiple enzymes acting on the substrate impacted the results, it is worth highlighting that these results might be the average of one or more isoenzymes. It is also possible that given our sample size (seven isolates and three enzymes), more differences in heat capacity may have been observed if we had increased the diversity and number of the isolates and enzymes in the experiment. Furthermore, specific experimental conditions, such as pH, could potentially alter the temperature-response curve. Determining how heat capacity varies phylogenetically for different enzymes is an important avenue for future research.

### Exercising Caution for Arrhenius Estimates of Temperature Sensitivity

Despite clear evidence of MMRT’s statistical superiority to Arrhenius in this experiment, we found that at the lower temperature ranges (i.e., 4–25 and 4–35°C) Arrhenius estimations were not necessarily poor. However, we still recommend that future estimations of temperature sensitivity for soil microbial enzymes that apply the Arrhenius equation use caution for the following reasons. First, we found that *E*_A_ values varied significantly with the range in which they were evaluated, making them an unreliable metric to use for comparisons across studies. These results are corroborated by Pawar et al. (2016), who tested 1,085 temperature-response curves from a variety of organisms and systems and determined that the calculated *E*_A_ value is an artifact of the temperature range, spread of temperatures measured, and where the temperature range falls. In order for temperature sensitivity to be used as a common currency of discussion and incorporated as a microbial trait, relationships should not be a function of different measurement methods. Second, even if Arrhenius is comparable to MMRT in a narrow temperature range, *E*_A_ fails to capture key phenomenological features of temperature sensitivity in soil biological systems. Other non-linear models have also been shown to give suitable empirical fits to the temperature dependence of enzyme activity (Peterson et al., 2004; Del Grosso et al., 2005; Daniel and Danson, 2013; Corkrey et al., 2014), but MMRT not only fits well empirically, but is derived from thermodynamic theory and thus has an underlying theoretical basis. Thus, even if *E*_A_ continues to be used in the future, *E*_A_ values should not be taken as true indicators of temperature sensitivity, at least for soil extracellular enzymes.

### Conceptual Framework

For nearly a decade, scientists have recognized the importance of using microbial traits as a framework for predicting ecosystem response to climate change (Green et al., 2008; Wallenstein and Hall, 2012). Many of these studies make predictions about how microbial traits (e.g., nutrient use efficiency) respond across a gradient of temperatures (Rinnan et al., 2009; Dell et al., 2011; Wallenstein and Hall, 2012). In this study we argue that temperature sensitivity is not only a measure of how biological traits respond across a gradient of temperatures, which is how it is typically characterized, but also that temperature sensitivity is an inherent biological trait. In light of this interest and our results, we developed a new conceptual model that develops a more precise definition of temperature sensitivity and organizes the factors that can lead to variation in temperature sensitivity itself.

**FIGURE 4:**
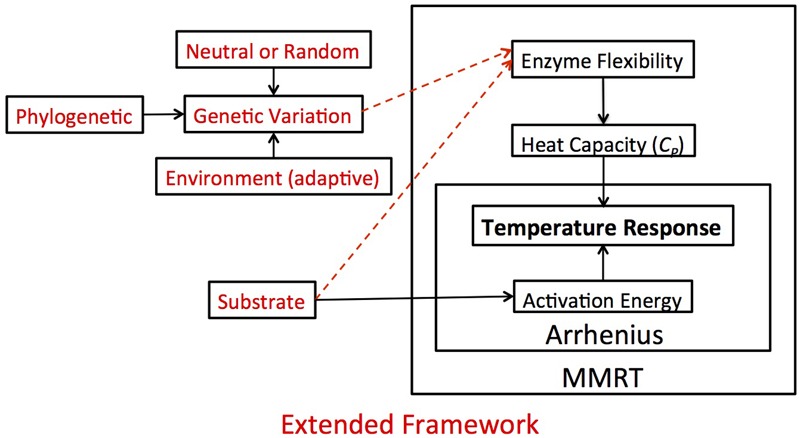
**Theoretical framework and hypotheses.** In this figure, we demonstrate how the Arrhenius and MMRT frameworks fit together conceptually and how that impacts our view of the temperature response, which in our case refers to the reaction rate of extracellular enzymes. Under the Arrhenius framework, activation energy is the singular factor driving the apparent temperature response. MMRT expands upon this framework, suggesting that the temperature response is a function of the heat capacity of an enzyme, which is related to the enzyme’s flexibility. While there is already evidence for substrate type influencing activation energy (solid arrow connecting substrate to activation energy), in this experiment (red lettering) we extend this framework and hypothesize that the enzyme flexibility is also a product of the substrate type and genetic variation among different enzymes (dashed arrows). We posit that this overarching framework could be applied in a variety of situations relating to the temperature response.

In our framework, we first consolidated the Arrhenius and MMRT definitions of temperature sensitivity. Under the Arrhenius equations, activation energy is the singular factor driving the apparent temperature response (**Figure 4**). But, this violates laws of thermodynamics with regards to biological systems because of the large molecular size of enzymes characterized by large heat capacities impacting the temperature response (Arcus et al., 2016). MMRT expands thermodynamic theory initiated with Arrhenius by incorporating heat capacity as part of the temperature response (**Figure 4**). Implicit within the MMRT theory is that the Δ$C_{p}^{‡}$ is a function of enzyme flexibility and thus Δ$C_{p}^{‡}$ varies among enzymes (Schulte, 2015; Arcus et al., 2016). Given existing evidence for substrate type influencing activation energy (Davidson and Janssens, 2006), our outline of temperature sensitivity includes potential for enzyme flexibility to be a product of the substrate upon which the enzyme acts as well as the genetic variation among different enzymes (**Figure 4**). In this experiment, we tested if these additional factors (i.e., substrate type and genetic variation) impacted the temperature sensitivity by measuring heat capacity as a proxy for enzyme flexibility and found strong evidence for heat capacity varying by both enzyme and isolate type.

Based on this expanded framework of temperature sensitivity, we propose that use of Δ$C_{p}^{‡}$ and TS_max_ will give a more comprehensive basis to describe “temperature sensitivity” than *Q*_10_ or *E*_A_. *Q*_10_ gives a false sense that a single constant can characterize the temperature sensitivity of a system (Davidson and Janssens, 2006). In order to overcome this obvious discrepancy authors using *Q*_10_ often present multiple temperature sensitivity values at different temperature ranges for a given system, leading to results that are often difficult to compare. Conceptually, we consider temperature sensitivity to be the change in velocity per change in temperature (*dV/dT*). Unlike *Q*_10_, *E*_A_ can be used as a summary term to capture temperature sensitivity of a system; this is effective because Arrhenius predicts a monotonic increase in rate with temperature. Since MMRT captures the inherently non-monotonic response of enzyme-catalyzed reactions, a single variable cannot fully capture the temperature sensitivity from the MMRT curve as is done by *E*_A_ in the Arrhenius equation. Thus the use of TS_max_ and *T*_opt_ provide two practical metrics to characterize this non-linear response of temperature sensitivity to temperature, although for modeling purposes Δ$C_{p}^{‡}$ and other thermodynamic parameters (i.e., Δ$S_{\mathrm{T0}}^{‡}$ and Δ$H_{\mathrm{T0}}^{‡}$) are sufficient to explicitly predict reaction rates with temperature. Temperature optimum values are also commonly reported in the literature for extracellular enzymes (Huston et al., 2000; Daniel et al., 2001; Peterson et al., 2004; Eijsink et al., 2005), but are typically quite high and perhaps not biologically relevant. We argue that TS_max_ is actually a more important term to consider than *T*_opt_ because TS_max_ describes where the greatest change in rate occurs and it typically falls within environmentally relevant temperature ranges (**Table 1**). Consequently, by focusing on TS_max_ we capture the area of the temperature-reaction curve that will have the greatest impact on rates of nutrient cycling and greenhouse gas production. Characterizing temperature sensitivity with these unifying parameters gives us an avenue to incorporate temperature sensitivity into traits-based microbial models.

Our new framework suggests a number of future lines of inquiry. One immediate question is: how broadly does temperature sensitivity vary under this new definition of temperature sensitivity? If temperature sensitivity of different enzymes, microbes, or communities exhibit vastly different Δ$C_{p}^{‡}$ and TS_max_ values then this might impact current calculations of soil C and N dynamics. As the climate warms, does this inherent temperature sensitivity adapt or acclimate? What are the evolutionary constraints on rate of evolution and how is the overall temperature sensitivity value impacting by different groups of organisms? What other factors besides enzyme type and the microbe from which it was produced might impact enzyme flexibility? We hope that future research will be conducted in many of these avenues to elucidate mechanisms controlling temperature sensitivity of enzymes and determine what impact this has on communities, ecosystems, and nutrient cycling in soils.

## Author Contributions

CA, JvF, PB, and MW developed the original concepts. CA and PB designed and performed the experiments. MW and CA contributed materials. CA conducted data analysis with significant involvement from JvF and NJ. CA wrote the manuscript with help from JvF, PB, MW, and NJ.

## Conflict of Interest Statement

The authors declare that the research was conducted in the absence of any commercial or financial relationships that could be construed as a potential conflict of interest.
