# Supplementary material for: Temperature Sensitivity as a Microbial Trait Using Parameters from Macromolecular Rate Theory
Source: Front Microbiol. 2016 Nov 17;7:1821. doi: 10.3389/fmicb.2016.01821 (PMC5112240; doi:10.3389/fmicb.2016.01821)
Supplement: Supplementary file 1 [file Table_1.DOCX]

**SUPPLEMENTARY TABLE S1** |Akaike information criterion corrected for a finite sample size (AICc) and *R*^2^ values for each model × isolate × enzyme combination. The MMRT values reported are for the full 4-60°C temperature range, while all three temperature ranges are reported for Arrhenius.

|  |  | MMRT | | Arrhenius 4-60°C | | Arrhenius 4-35°C | | Arrhenius 4-25°C | |
| --- | --- | --- | --- | --- | --- | --- | --- | --- | --- |
| Enzyme | Isolate | AICc | R^2^ | AICc | R^2^ | AICc | R^2^ | AICc | R^2^ |
| BG | Aci | 63.54 | 0.62 | 97.38 | 0.22 | 10.36 | 0.40 | 7.45 | 0.39 |
|  | Cit | -0.71 | 0.94 | 105.69 | 0.47 | 41.32 | 0.82 | 58.69 | 0.81 |
|  | Com | 65.62 | 0.58 | 88.42 | 0.32 | 65.42 | 0.40 | 79.04 | 0.38 |
|  | Ent | 3.42 | 0.94 | 118.95 | 0.14 | -13.52 | 0.97 | -6.32 | 0.97 |
|  | Fla | -30.38 | 0.97 | 105.27 | 0.51 | 14.30 | 0.92 | 20.72 | 0.92 |
|  | Pse | 73.32 | 0.55 | 94.03 | 0.30 | 70.72 | 0.34 | 98.53 | 0.33 |
| LAP | Aci | 11.87 | 0.96 | 129.19 | 0.45 | -2.28 | 0.97 | -2.06 | 0.97 |
|  | Bac | 24.69 | 0.97 | 526.13 | 0.90 | -25.26 | 0.99 | -23.92 | 0.99 |
|  | Cit | -4.67 | 0.96 | 110.94 | 0.54 | 14.02 | 0.95 | 25.17 | 0.95 |
|  | Com | 4.62 | 0.95 | 96.74 | 0.60 | -57.48 | 0.99 | -52.69 | 0.99 |
|  | Ent | -29.40 | 0.98 | 108.18 | 0.59 | 22.37 | 0.93 | 37.94 | 0.93 |
|  | Fla | 14.13 | 0.93 | 102.20 | 0.53 | 38.37 | 0.87 | 54.45 | 0.86 |
|  | Pse | 14.16 | 0.91 | 77.15 | 0.60 | 12.22 | 0.91 | 13.92 | 0.90 |
| PHOS | Aci | 65.40 | 0.62 | 94.15 | 0.30 | 57.43 | 0.59 | 75.01 | 0.58 |
|  | Bac | -38.32 | 0.97 | 93.07 | 0.81 | 4.56 | 0.93 | 21.31 | 0.93 |
|  | Cit | -31.69 | 0.97 | 55.21 | 0.80 | 6.98 | 0.92 | 21.43 | 0.92 |
|  | Ent | -85.80 | 0.99 | 64.58 | 0.80 | -21.68 | 0.97 | 0.56 | 0.97 |
|  | Fla | 67.49 | 0.72 | 97.18 | 0.49 | 67.79 | 0.57 | 93.49 | 0.56 |
|  | Pse | 56.54 | 0.59 | 77.34 | 0.37 | 55.42 | 0.48 | 83.56 | 0.47 |
